# Regulation of Ascorbate Accumulation and Metabolism in Lettuce by the Red:Blue Ratio of Continuous Light Using LEDs

**DOI:** 10.3389/fpls.2020.00704

**Published:** 2020-05-29

**Authors:** Lingyan Zha, Wenke Liu, Qichang Yang, Yubin Zhang, Chengbo Zhou, Mingjie Shao

**Affiliations:** ^1^Institute of Environment and Sustainable Development in Agriculture, Chinese Academy of Agricultural Sciences, Beijing, China; ^2^Key Laboratory of Energy Conservation and Waste Management of Agricultural Structures, Ministry of Agriculture and Rural Affairs, Beijing, China; ^3^Institute of Urban Agriculture, Chinese Academy of Agriculture Science, Chengdu, China

**Keywords:** ascorbic acid, enzyme activity, expression level, light quality, regulatory mechanism

## Abstract

Ascorbate (AsA), an antioxidant that cannot be synthesized and stored by the human body, plays an essential role in the proper functioning of both plants and humans. With the goal of increasing the AsA level in lettuce, the effects of different ratios of red (R) to blue (B) light (75R:25B, 50R:50B, and 25R:75B) on AsA pool sizes as well as the transcript levels and activities of key enzymes involved in AsA metabolism were constantly monitored for 12 days under continuous light (200 μmol⋅m^–2^⋅s^–1^) from LEDs. The results showed that lettuce biomass was positively correlated with the ratio of red light, while the AsA pool size had a positive correlation with the ratio of blue light during the whole experiment. The 25R:75B treatment increased the expression of genes involved in AsA biosynthesis (*GMP*, *GME*, *GGP*, *GPP*, *GLDH*) and regeneration (*APX*, *MDHAR*, *DHAR*, and *GR*) on day 3 but only significantly elevated the activities of enzymes involved in AsA regeneration (APX, MDHAR, DHAR, and GR) subsequently. AsA regeneration enzymes (MDHAR, DHAR and GR) had greater correlations with the AsA level than the AsA synthesis enzyme (GLDH). Thus, it is concluded that a high ratio of blue light elevated the AsA level mainly by promoting AsA regeneration rather than biosynthesis. Taken together, altering the red:blue ratio of continuous light from high to low before harvest is recommended for lettuce cultivation to achieve both high yield and high quality.

## Introduction

Ascorbate, the reduced form of vitamin C, is well recognized as one of the most pivotal antioxidants in plants (Gallie, 2013; Ntagkas et al., 2018). It has multiple essential functions in the regulation of many physiological processes in plants (Davey et al., 2000). The metabolic pathway of AsA in plants has been well established since Wheeler et al. (1998) proposed the main AsA biosynthetic pathway: the L-galactose pathway (D-mannose pathway). In this pathway, D-glucose is catalyzed to AsA by a series of enzymes, including several key enzymes: GDP-d-mannose pyrophosphorylase (GMP), GDP-d-mannose 3′5-epimerase (GME), GDP-L-galactose phosphorylase (GGP), L-galactose-1-phosphate phosphatase (GPP), and L-galactono-1,4-lactone dehydrogenase (GLDH), which catalyzes the final step. After AsA is synthesized, it is oxidized to monodehydroascorbate (MDHA) by ascorbate peroxidase (APX), and MDHA is then spontaneously disproportionated to dehydroascorbate (DHA). Fortunately, MDHA and DHA can be reduced back to AsA by monodehydroascorbate reductase (MDHAR) and dehydroascorbate reductase (DHAR), respectively. The electron donors of these two enzymes are NADPH and glutathione, respectively, and the latter electron donor is recovered from oxidized glutathione by glutathione reductase (GR) (Ntagkas et al., 2018).

Sufficient AsA level is also vital for maintaining the proper physiological function of the human body (Ntagkas et al., 2018). However, unlike in plants, although AsA is indispensable for the human body, it cannot be synthesized and stored by humans. Thus, AsA in the human body can only be provided by the diet, especially by vegetables. Compared with vegetables cultivated in open fields, those cultivated in protected horticulture, which occupy a large part of the vegetable market, often have relatively lower AsA levels. Furthermore, with the improvement of human awareness of food safety and health, low nutritional quality has become a reason for consumers to resist the purchase and consumption of vegetables that cultivated in protected horticulture. This resistance has seriously restricted the development and application of protected horticulture, including greenhouses and plant factories. Increasing effective light is a direct and effectual way to improve the AsA levels, as the relatively weak irradiance in protected horticulture is one of the main reasons for the low AsA level of vegetable cultivated in protected horticulture (Massot et al., 2010). Continuous light is an operative way to increase effective light by maximizing light period in protected horticulture. Although numerous studies have shown that continuous light induced severe leaf damage of some sensitive plant species, such as tomato, there were also some species could tolerance continuous light and showed positive responses (Sysoeva et al., 2010; Velez-Ramirez et al., 2011). Our previous study indicated that continuous light improved both the yield and AsA level of hydroponic lettuce without causing any leaf injury and dysfunction compared with lettuce grown under a normal photoperiod (16 h light/8 h dark) (Zha et al., 2019b). Optimize light parameters of continuous light could further elevate AsA level, as light is the most vital environment factor affecting AsA level (Ntagkas et al., 2018). We have discovered that increasing the light intensity of continuous light can further improve the AsA level, but consumed more electric energy at the same time (Zha et al., 2019a). Improving AsA levels by light quality regulation might be more preferable from the energy perspective.

The effect of different light qualities on AsA content has been studied in many plants, including lettuce (Chen et al., 2016), basil and parsley (Samuolienë et al., 2016), and citrus fruits (Zhang et al., 2015). The majority of studies have shown that the short-wavelength spectrum (e.g. UV-A, blue light) is more conducive to increasing the AsA content compared to long-wavelength spectrum (Zhang et al., 2015; Dutta Gupta, 2017). However, the opposite results have also been reported. For example, Ma et al. (2014) found that red light irradiation after harvest could effectively suppress the reduction of AsA levels in broccoli, while blue light could not. Such inconsistent results indicate that the effect of light quality on AsA level is not certain; it is influenced by many factors, such as plant species, light intensity, or other environmental factors (Dutta Gupta, 2017). To date, most previous studies have focused on the response of the AsA quantity to light quality, but the mechanisms and physiological basis of light quality regulation on AsA metabolism has relatively been less studied. Zhang et al. (2015) found that blue light upregulated the expression of AsA biosynthetic genes and AsA regeneration genes to increase the AsA content compared with that under dark conditions. Similar to Zhang, several researchers explained the mechanism of light quality or other environmental factors regulating AsA accumulation by investigating changes in gene expression (Mastropasqua et al., 2012; Massot et al., 2013; Ma et al., 2014; Zhang et al., 2015). However, gene expression is sometimes not consistent with AsA content (Pignocchi et al., 2003; Bartoli et al., 2006), as translation, posttranscriptional regulation, and other processes occur after transcription. In fact, mRNA level of a particular gene not often has direct correlation with its protein content in plants (Veìlez-Bermuìdez and Schmidt, 2014). Therefore, to understand the process by which light quality regulates AsA metabolism, it is necessary to explore at both the levels of transcription and enzyme activity.

Red and blue lights are recognized as the most important spectrum for plant growth because they are not only the major light source for photosynthesis but they also regulate many morphogenetic responses in plants through photoreceptors (Xu et al., 2012; Lin et al., 2013), but monochromatic red or blue light is not conducive to plant growth. Currently, the main light sources in protected horticulture are red and blue LED lights, thus it is more practical to explore the influence of the red:blue ratio on AsA accumulation and metabolism. Lettuce is the main vegetable species cultivated in plant factories and has abundant ascorbate, which is equivalent to the level in some fruits (e.g. tomato). To elevate the potential of the promoting effect of continuous light on ascorbate accumulation in lettuce, the red:blue ratio of the continuous light needs to be optimized. In the present study, the effects of the red:blue light ratio on ascorbate accumulation and the activities and gene expression of enzymes related to ascorbate metabolism were investigated under continuous light using LEDs. We hypothesize that regulation of red:blue light ratio on AsA involves several regulatory points in its metabolism pathway at both transcriptional and enzymatic level, and correlate with oxidative stress under continuous light. The objective of this study is to provide new lighting strategies to enhance AsA level in lettuce and explore the mechanism of red and blue light regulating on AsA metabolism.

## Materials and Methods

### Plant Materials and Light Treatments

**FIGURE 1:**
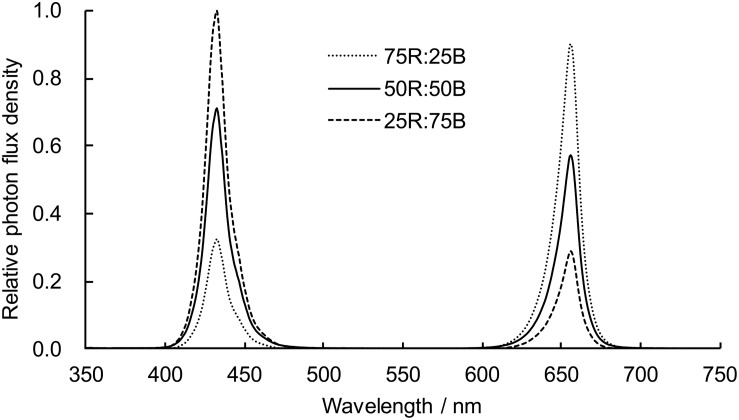
Light spectra of light treatments.

Lettuce plants were hydroponically cultivated in an environment-controlled plant factory with atmospheric carbon dioxide, 23 ± 3°C air temperature, and 50–60% relative humidity. Lettuce seeds were sown on soaked sponges (2.5 × 2.5 × 2.5 cm) on plastic germination trays and then germinated under white LED (200 μmol⋅m^–2^⋅s^–1^, 16/8 h) light after sprouting. Two weeks later, the seedlings were transplanted from the germination trays to a recirculating hydroponic culture system equipped with red and blue LED light panels. To make the lettuce seedlings grow evenly and adapt to the new system, all seedlings were exposed to uniform light conditions (75R:25B, 200 μmol⋅m^–2^⋅s^–1^, 16/8 h) for 10 days. Then, seedlings were randomly divided into three groups (39 plants for each group) to receive continuous light (200 μmol⋅m^–2^⋅s^–1^) of different light qualities: 75R:25B, 50R:50B, and 25R:75B (Table 1 and Figure 1). Modified Hoagland nutrient solution (pH≈5.8; EC≈1.6 dS⋅m^–1^) was applied for plant cultivation and was circulated for 60 min every day. The light intensity was measured by a light sensor logger (Li-1500) and a quantum sensor (LI-190R, Lincoln, NE, United States), and the light spectra were confirmed by a spectroradiometer (Avaspec-2048CL, Avates, Apeldoorn, Netherlands).

**TABLE 1:** Light spectrum, light intensity, and photoperiod of each treatment at each growth stage of lettuce. W: white LED light, R: red LED light, B: blue LED light.

Treatments	Germination stage (15 days)	Acclimation stage (10 days)	Treatment stage (12 days)
75R:25B	W: 200 μmol⋅m^–2^⋅s^–1^ 16/8 h	R: 150 μmol⋅m^–2^⋅s^–1^ B: 50 μmol⋅m^–2^⋅s^–1^ 16/8 h	R: 150 μmol⋅m^–2^⋅s^–1^ B: 50 μmol⋅m^–2^⋅s^–1^ 24/0 h
50R:50B			R: 100 μmol⋅m^–2^⋅s^–1^ B: 100 μmol⋅m^–2^⋅s^–1^ 24/0 h
25R:75B			R: 50 μmol⋅m^–2^⋅s^–1^ B: 150 μmol⋅m^–2^⋅s^–1^ 24/0 h

### Sampling and Measurements of Growth Parameters

The samples used for the physiological and gene expression determinations were collected at 21:00 (the end of the light period at the germination and acclimation stages) every 3 days (0, 3, 6, 9, and 12 days) after the start of the treatments. Leaves without petioles from four lettuce plants were sampled from each treatment at each sampling time and were kept as four biological replicates. The collected leaves were immediately frozen in liquid nitrogen and stored in an ultralow temperature freezer (−80°C) until analysis. For the measurement of growth parameters, another five plants were sampled on day 12 after the beginning of the treatment. The shoots and roots of the lettuce plants were separated to determine the fresh weight (FW), respectively. The main petioles of all leaves were then removed to accurately measure the leaf FW and leaf area with an area meter (LI-3100, Li-Cor Biosciences, Lincoln, NE, United States). Specific leaf FW was the ratio of leaf FW to leaf area. After these determinations, all shoots and roots were dried in an oven at 80°C for the determination of shoot and root dry weights (DW), which were used to calculate the root/shoot ratio and shoot DW/FW ratio.

### Ascorbate Pool Size Assays

The total ascorbate (T-AsA) and AsA concentrations of the four biological replicates were determined by UPLC according to the methods of Spiìnola et al. (2012) and Campos et al. (2009) with some adaptations. Frozen leaf tissue (0.1 g) was homogenized in 1 mL precooled extractant solution that contained 1.5% (w/v) metaphosphoric acid, 4% (v/v) acetic acid, and 0.5 mM EDTA. After centrifugation (15000 × *g*, 4 °C, 15 min), the supernatant was filtered through PTFE filters (0.22 μm) and collected to assay the concentration of T-AsA and AsA. For the T-AsA content determination, 50 μL supernatant was mixed with filtered (0.22 μm PTFE filters) dithiothreitol (10 μL, 750 mM), Tris (190 μL, 275 mM), and sulfuric acid (50 μL, 0.4 M) and then incubated for 30 min at 25 °C. The reaction mixture was analyzed by an Acquity UPLC system (Waters Corp, United States) with an Acquity UPLC HSS T3 column (2.1 × 100 mm, 1.8 μm, Waters). The column was eluted with 0.1% (v/v) formic acid with a flow rate of 0.25 mL⋅min^–1^. The absorbance at 245 nm was monitored by a Waters Acquity UPLC photodiode array (PDA) (Waters Corp, United States) detection system. The AsA content was analyzed in a similar manner except that 10 μL deionized H_2_O was substituted for the dithiothreitol. The DHA content was calculated as the difference between the T-AsA and AsA contents.

### Enzyme Activity Assays

The extraction of GalLDH (EC 1.3.2.3), APX (EC 1.11.1.11), MDHAR (EC 1.6.5.4), DHAR (EC 1.8.5.1), and GR (EC 1.8.1.7), as well as the determination of the activities of these enzymes, have been described in detail previously (Zha et al., 2019a). Four biological replicates were used to perform the enzyme activity assay.

### Hydrogen Peroxide and Malondialdehyde Content Assays

The hydrogen peroxide (H_2_O_2_) and malondialdehyde (MDA) contents of the four biological replicates were assayed by UV-VIS spectrophotometer (Shimadzu UV-1800, Kyoto, Japan) according to the method of Brennan and Frenkel (1977) and Yang et al. (2010), respectively. H_2_O_2_ was extracted from 0.1 g fresh frozen leaf tissue by homogenization with 1 mL precooled acetone. After centrifugation (10,000 *g*, 20 min, 4°C), 1 mL supernatant was mixed with 0.1 mL of 10% (v/v) titanium sulfate and 0.2 mL ammonia and centrifuged at 4,000 *g* for 10 min at 25°C. The precipitate was then dissolved in 1 mL 2 M H_2_SO_4_ to measure the absorbance at 412 nm. The MDA was extracted from 0.1 g fresh-frozen leaf tissue by homogenization with 1 ml cold 10% trichloroacetic acid. After centrifugation (15,000 *g*, 10 min, 4°C), 0.5 mL supernatant and 0.5 mL 0.6% thiobarbituric acid were mixed and boiled at 100 °C for 20 min and then quickly cooled to room temperature. The reaction mixture was centrifuged at 15,000 *g* for 10 min to collect the supernatants, which were used to measure the absorbance at 450, 532, and 600 nm.

### Total RNA Extraction and RT-qPCR Analysis

The total RNA of the lettuce leaves was extracted by the RNAprep Pure Plant Plus Kit (DP441, Tiangen Biotech Co., Ltd., Beijing, China) according to the manufacturer’s instructions. The quality and concentration of total RNA was determined by an ultra-micromole plate spectrophotometer (TECAN, Infinite M200 Pro, Switzerland). 2 μg total RNA was used for reverse transcription using the FastKing RT kit (KR116, Tiangen Biotech Co., Ltd., Beijing, China) in a 20 μL reaction and the reverse transcription PCR was conducted according to the instructions. Quantitative RT-PCR analysis was performed using a multicolor real-time PCR detection system (Bio-Rad, Hercules, CA, United States). For the qPCR, each reaction included 9 μL 100X diluted cDNA, 1 μL primer (initial concentration was 10 μM) and 10 μL PCR buffer including SYBR green. The optimized program of the PCR protocol included initial denaturation at 95°C for 5 min, followed by 39 cycles of 95°C for 10 s, 60°C for 30 s, and 72°C for 30 s. The primers sequences are presented in Supplementary Table S1. 18S rRNA was used as housekeeping gene. To compare the gene expression levels under varying red:blue light ratios and different days of continuous light, the gene expression level of every measured enzyme in the lettuce leaves before the continuous light was quantified as 1. For relative quantification, the 2^–(ΔΔ*Ct)*^ method was used according to Livak and Schmittgen (2001). Three independent biological replicates were used for each treatment.

### Statistical Analysis

Growth (*n* = 5) and physiological (*n* = 4) parameters were subjected to one-way ANOVA and two-way ANOVA, respectively, by SPSS 18.0 (International Business Machines Corporation). For two-way ANOVA, light treatment and days of light treatments were considering as two factors, and the results were list in Table 3. After variance analysis, Tukey’s test (*p* < 0.05) was used to make post-hoc multiple comparisons among means of different light treatments at each time point. Correlation and significance tests among AsA pool sizes and enzyme activities were calculated using the Pearson correlation coefficient with a two-tailed test. Principal component analysis (PCA) was performed by Canoco 5.0 (Microcomputer Power, Ithaca, NY, United States).

## Results

### Plant Growth

After 12 days of continuous light with different ratios of red:blue light, the shoot FW of lettuce increased significantly with increasing red:blue light ratio, and the differences in shoot FW between the 25R:75B and 75R:25B treatments reached a significant level (Table 2). Although the shoot DW and leaf area also showed a positive correlation with the red:blue light ratio, the differences in them between 50R:50B and 75R:25B treatments were greater than those between 25R:75B and 50R:50B, and there was no significant difference in these parameters among the treatments. The root FW, root DW, and root/shoot ratio of 75R:25B treatments were the highest and significantly greater than those of the other two treatments. Both the shoot DW/FW and specific leaf FW were the lowest under the 50R:50B treatment, but the differences among treatments were not significant.

**TABLE 2:** The shoot fresh weight (FW), shoot dry weight (DW), shoot DW/FW, root/shoot ratio, leaf area, and specific leaf FW of lettuce plants grown under continuous light (200 μmol⋅m^–2^⋅s^–1^) with different red:blue ratios.

Treatments	Shoot FW (g)	Shoot DW (g)	Shoot DW/FW (%)	Root FW	Root DW	Root/shoot ratio	Leaf area (dm^2^)	Specific leaf FW (g/dm^2^)
25R:75B	88.5 b	2.98 a	3.40 a	8.5b	0.44b	0.149 b	11.10 a	4.13 a
50R:50B	94.2 ab	3.02 a	3.25 a	8.7b	0.46b	0.151 b	11.75 a	4.07 a
75R:25B	99.5 a	3.55 a	3.36 a	11.0a	0.62a	0.187 a	12.70 a	4.23 a

*The samples were taken on the 12th day after treatment. Values are the means of five replicates. Different letters indicate significant differences between different treatments at *p* < 0.05 according to the Tukey test.*

**TABLE 3:** *P*-values of the two-way ANOVA for the effects of light treatments (Light) and days of light treatments (Day) on the physiological parameters.

	T-AsA	AsA	DHA	AsA/T-AsA	GalLDH	APX	MDHAR	DHAR	GR	H_2_O_2_	MDA
P_(Light)_	**	**	*	ns	ns	**	**	**	**	**	*
P_(Day)_	ns	ns	ns	ns	*	**	**	ns	**	**	**
P_(Light× Day)_	ns	ns	ns	ns	ns	ns	*	ns	ns	*	ns

Non-significant (ns) indicates *p* > 0.05, * and ** indicate significant at *p* ≤ 0.05 and 0.01, respectively.

### Ascorbate Pool

**FIGURE 2:**
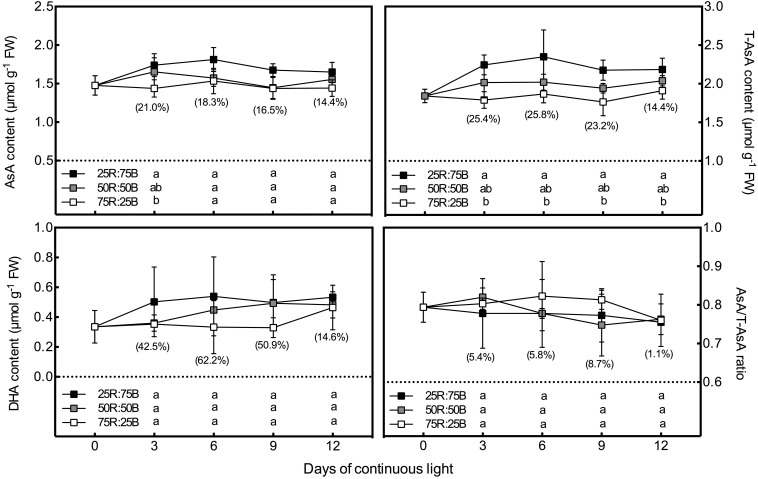
Effects of the red:blue light ratio on the total ascorbate (T-AsA), ascorbate (AsA) and dehydroascorbate (DHA) contents as well as the AsA/T-AsA ratio in lettuce leaves under continuous light (200 μmol⋅m^– 2^⋅s^– 1^). Values and bars represent the means of four replicates ± SD. Different letters in the same column indicate significant differences at the *p* < 0.05 level according to Tukey test. Data in brackets were the increasing rate of the maximum value to the minimum value on the same day.

In general, under continuous light with different red:blue light ratios, the T-AsA, AsA, and DHA contents in lettuce leaves showed positive correlations with the ratio of blue light (Figure 2). Compared to the 75R:25B treatment, the T-AsA content of 25R:75B increased by 14.4∼25.8%. The differences in T-AsA content in lettuce between 25R:75B and 75R:25B treatments reached a significant level during the whole experiment. There was no significant difference in T-AsA content between 50R:50B and 75R:25B as well as 25R:75B and 50R:50B. The differences in T-AsA content between 25R:75B and 50R:50B were greater than those between 50R:50B and 75R:25B. There was no significant difference in the AsA and DHA contents among treatments except on day 3, when the AsA content under the 25R:75B treatment was significantly higher than that under the 75R:25B treatment. In addition, the change tendencies of the AsA levels in lettuce under the 25R:75B and 75R:25B treatments remained relatively stable. However, under the 50R:50B treatment, the AsA and DHA contents continuously decreased and increased from day 3 to day 9, respectively, which resulted in the decline of the AsA/T-AsA ratio at the same time. The AsA/T-AsA ratio of 75R:25B treatment was the highest among treatments on day 6 and 9, but red:blue light ratio had no significant effect on AsA/T-AsA ratio (Table 3).

### Ascorbate Biosynthesis

**FIGURE 3:**
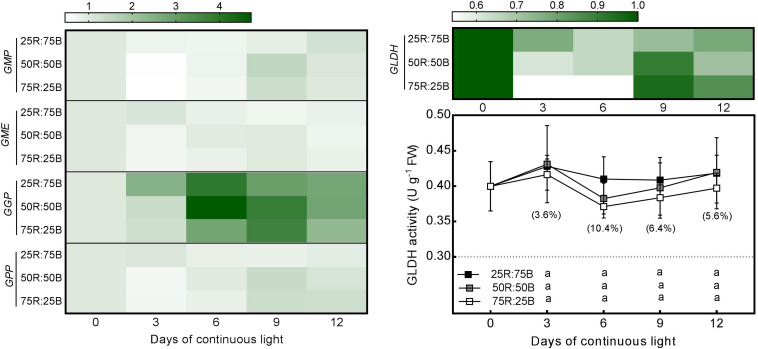
Effects of the red:blue light ratio on the transcript level of enzymes involved in the AsA biosynthesis pathway and activity of L-galactono-1,4-lactone dehydrogenase (GalLDH) in lettuce leaves under continuous light (200 200 μmol⋅m^– 2^⋅s^– 1^). Transcript level data are the mean values of three replications. Enzyme activity data are the mean values ± SD of four replications. Different letters indicate significant differences between different red:blue light ratio treatments at *p* < 0.05 according to Tukey test. Data in brackets were the increasing rate of the maximum value to the minimum value on the same day.

Changes in the expression of several critical AsA biosynthetic genes (*GMP*, *GME*, *GGP*, *GPP*, and *GLDH*) and the activity of the last synthetase (GLDH) in response to continuous light at different red:blue light ratios were analyzed in lettuce (Figure 3). The expression of the abovementioned genes in lettuce leaves was upregulated by the 25R:75B treatment on day 3, but they nearly increased 2∼3-fold from day 3 to day 9 under the other two treatments, resulting in a greater transcript level than that under the 25R:75B treatment on day 9. Unlike gene expression, GLDH activity presented little response to the red:blue light ratio under continuous light. There was no significant difference in GLDH activity among treatments.

### Ascorbate Oxidation and Reduction (Ascorbate-Glutathione Cycle)

**FIGURE 4:**
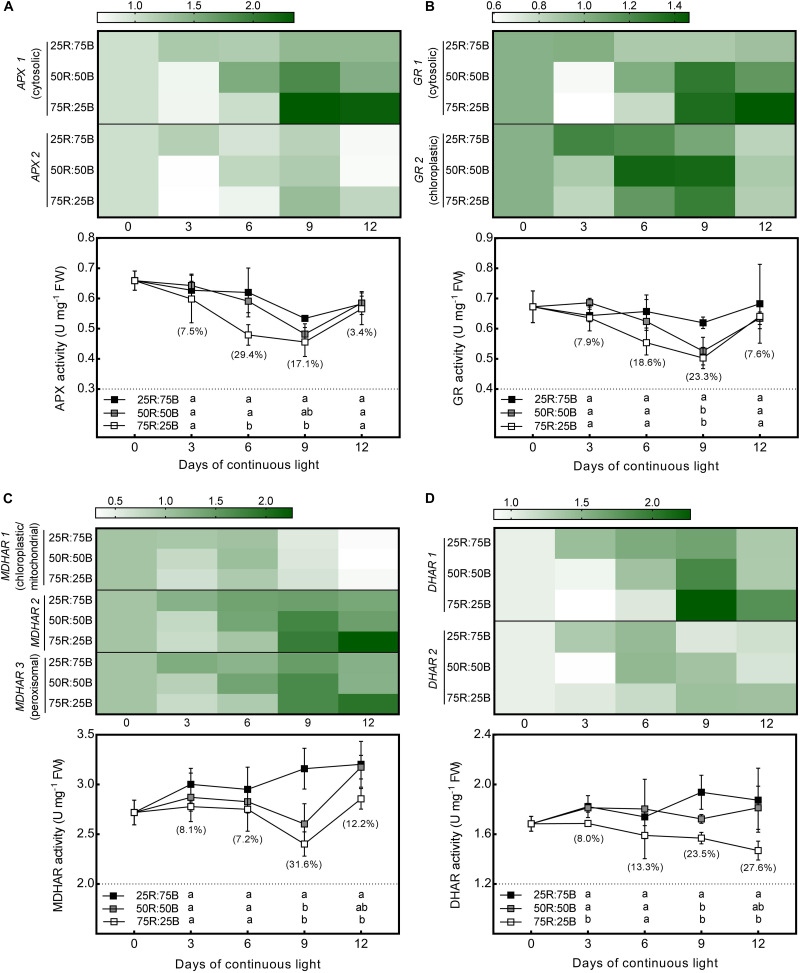
Effects of the red:blue light ratio on the transcript level and activity of APX **(A)**, GR **(B)**, MDHAR **(C)**, DHAR **(D)** in lettuce leaves under continuous light (200 μmol⋅m^– 2^⋅s^– 1^). Transcript level data are the mean values of three replications. Enzyme activity data are the mean values ± SD of four replications. Different letters indicate significant differences between different red:blue light ratio treatments at *p* < 0.05 according to Tukey test. Data in brackets were the increasing rate of the maximum value to the minimum value on the same day.

As shown in Figure 4, the changes in the gene expression levels and activities of enzymes involved in AsA oxidation and reduction in response to the red:blue light ratio under continuous light were analyzed. Similar to the biosynthetic genes, all the genes we investigated that involved in AsA oxidation (*APX 1*, *APX 2*) and reduction (*DHAR 1*, *DHAR 2*, *MDHAR 1*, *MDHAR 2*, *MDHAR 3*, *GR 1*, and *GR 2*) were more highly expressed under the 25R:75B treatment on day 3. The transcript levels of *APX 1*, *APX 2*, *DHAR 1*, *MDHAR 2*, *MDHAR 3*, *GR 1*, and *GR 2* under the 50R:50B and 75R:25B treatments showed much greater increases than those under the 25R:75B treatment from day 3 to day 9; thus, the expression levels of these genes were lowest under the 25R:75B treatment on day 9.

The change tendencies of APX and GR activities with time and the differences in their activities among treatments were quite similar (Figures 4A,B). Activities of APX and GR in all treatments decreased gradually with time during the first 9 days. They decreased most rapidly under the 75R:25B treatment and slowest under the 25R:75B treatment. Therefore, the APX and GR activities had a positive correlation with the blue light levels on day 6 and day 9, and the differences in their activity between the 75R:25B and 25R:75B treatments reached a significant level. During the whole experimental period, the MDHAR activity of lettuce leaves grown under continuous light remained the lowest and the highest under the 75R:25B and 25R:75B treatments, respectively, and the difference between the LL and HL leaves reached a significant level on day 6 and day 9 (Figure 4C). The DHAR activity in the 75R:25B treatment also remained at the lowest level and was significantly lower than that in the 25R:75B treatment, except on day 6 (Figure 4D).

### H_2_O_2_ and MDA Content

**FIGURE 5:**
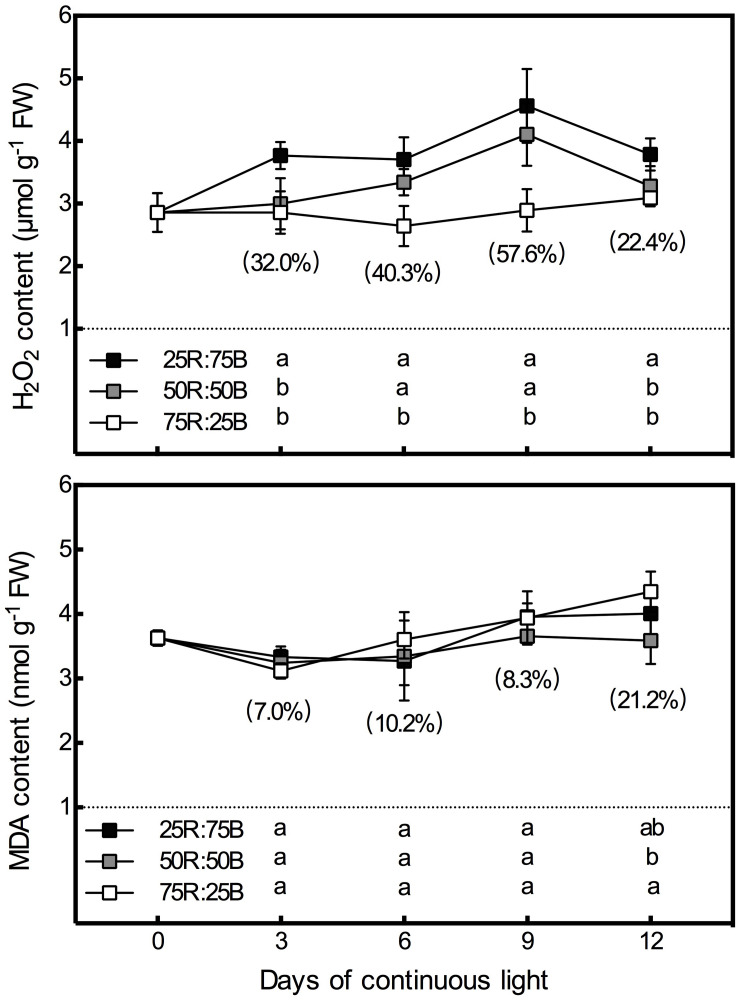
Effects of the red:blue light ratio on the H_2_O_2_ and MDA contents in lettuce leaves under continuous light (200 μmol⋅m^– 2^⋅s^– 1^) (200 μmol⋅m^– 2^⋅s^– 1^). Data are the mean values ± SD of four replications. Different letters indicate significant differences between different red:blue light ratio treatments at *p* < 0.05 according to Tukey test. Data in brackets were the increasing rate of the maximum value to the minimum value on the same day.

Figure 5 shows that there was a significant positive correlation between the H_2_O_2_ content in lettuce leaves and the ratio of blue light. The H_2_O_2_ content of the 25R:75B treatment was significantly higher than that of the 75R:25B treatment during the whole test period, and the difference in H_2_O_2_ between the 75R:25B and 50R:50B treatments reached a significant level on days 6 and 9. The content of H_2_O_2_ in the 25R:75B and 50R:50B treatments increased constantly with time during the first 9 days but decreased obviously from days 9 to 12. However, the H_2_O_2_ content was maintained at a relatively stable level under the 75R:25B treatment. Compared with H_2_O_2_, the red:blue light ratio had no significant influence on the MDA content during the first 9 days. As the MDA content under the 75R:25B treatment increased constantly from day 3 to day 12, it was significantly higher than that of the 50R:50B treatment on day 12.

### Correlation Analysis and Principal Components Analysis

**FIGURE 6:**
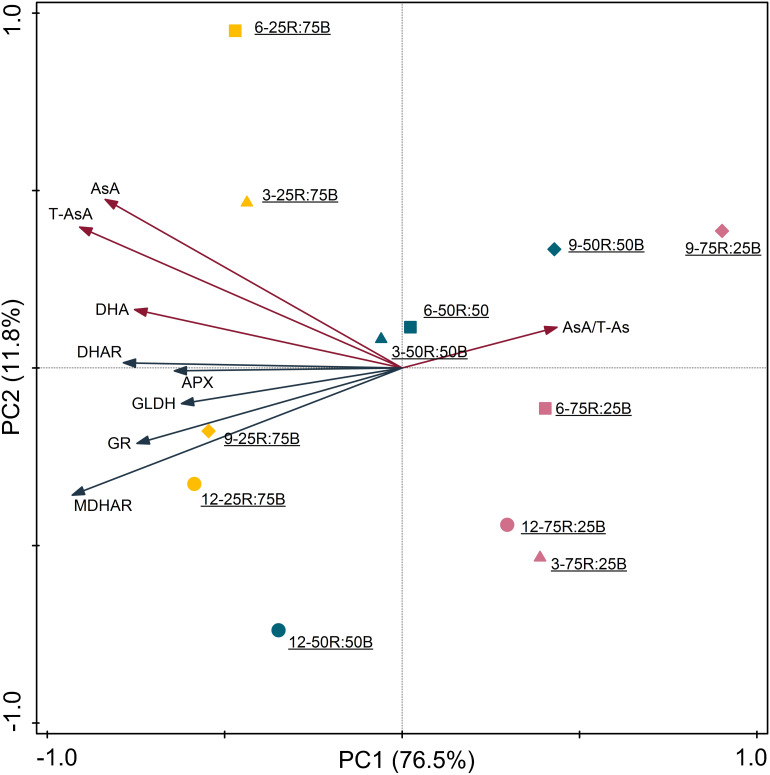
Principal component analysis of AsA pool sizes and enzyme activities in lettuce leaves under continuous light of different red:blue ratios (yellow symbol-25R:75B; blue symbol-50R:50B; red symbol-75R:25B) on different days (△–day 3; □–day 6, ♢–day 9,○–day 12). Biplot of the first two principal components (PC1,2) of the measured network topological properties.

The correlation coefficients among the indexes of AsA pool levels and enzymes activities, analyzed by Pearson’s correlation are listed in Table 4. Among the five enzymes involved in AsA metabolism, GLDH activity was not correlated with AsA pool levels, including T-AsA, AsA, and DHA. Activities of MDHAR, DHAR, and GR had significant correlations with both T-AsA and AsA levels, while APX activity only had a significant correlation with AsA level. Meanwhile, APX activity had an extremely strong correlation with GR activity. The biplot (Figure 6) of PCA results showed that AsA pool levels and enzymes activities were negatively related to PC1, while the AsA/T-AsA was positively related to PC1. In general, the points of different light treatments were mainly separated along PC1.

**TABLE 4:** Pearson’s correlation coefficients among the AsA pool sizes and enzyme activities in lettuce exposed to continuous light of different red:blue ratios.

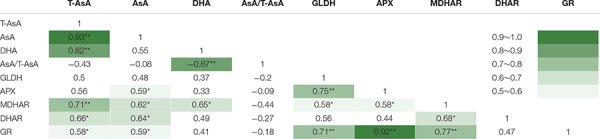

Correlation was significant at *p < 0.05; **p < 0.01. The deeper the color is, the greater the correlation coefficient.

## Discussion

### Continuous Light With a High Red:Blue Ratio Is Conducive to Lettuce Growth

Red light and blue light are the most important light spectra for plants, as their photosynthetic relative quantum efficiencies are much greater than those of other light spectra (McCree, 1972; Stutte, 2009). Many studies have focused on the effects of the red:blue light ratio on the growth of vegetables, including lettuce, which is the main plant species cultivated in plant factories. Although the optimal ratio of red:blue light for lettuce growth was not unified due to various varieties and environment factors, most studies have shown that light spectrum with a predominating portion of red light (e.g. 75∼90%) is more conducive to increasing lettuce biomass under a normal photoperiod (Cope et al., 2014; Wang et al., 2016). Our results indicated that this phenomenon also emerged under continuous light. This is because red light has higher relative quantum efficiency than blue light (McCree, 1972; Stutte, 2009), and photosynthetic capacity will be decreased when the proportion of blue light exceeds 50%, even though moderate blue light (e.g. 7%) is essential for preventing dysfunctional photosynthesis under pure red light (Hogewoning et al., 2010). The positive correlation between biomass and red light ratio partly related to leaf area which also positively correlated with red light ratio, since high level of blue light inhibits leaf area expansion (Bugbee, 2016). Root elongation is also inhibited by excessive blue light (Oyaert et al., 1999; Spalding and Folta, 2005), the root FW, root DW, and root/shoot ratio in the present study were significantly elevated by 75R:25B treatment. It has been reported that red light and blue light regulate root growth by affecting the distribution of phytchormones (Drozdova et al., 2001). Overall, continuous light with high ratio of red light can elevate lettuce photosynthetic efficiency and photosynthetic products synthesis, while promote transportation of photosynthetic products from shoot to root. Vigorous roots in turn supply more water and mineral nutrition for shoot growth.

### Continuous Light With a Low Red:Blue Ratio Is Conducive to AsA Accumulation in Lettuce

Previous studies have indicated that long-wavelength light (e.g. red light) is conducive to the growth of plants, while short-wavelength light (e.g. blue light, UV A) is conducive to the promotion of nutritional quality through stimulating the accumulation of secondary metabolites, such as ascorbate, flavonoids, and anthocyanins (Li and Kubota, 2009; Ren et al., 2014). Consistent with previous results, our results indicate that although lettuce gained greater biomass under the higher red:blue light ratio (75R:25B), the ascorbate pool was improved by the higher ratio of blue light (25R:75B) with the same total light intensity. The more effective impact of blue light than red light on enhancing the AsA content in the juice sacs of citrus fruits (Zhang et al., 2015) and oat leaves (Mastropasqua et al., 2012) has also been proposed. Some studies have shown that the opposite result also occurred under some conditions. For instance, Ma et al. (2014) found that red light was more effective than blue light in suppressing AsA reduction in postharvest broccoli. This might be related to the greater APX activity under blue light. Furthermore, some previous studies have suggested that photochromes and blue-light photoreceptors are involved in the regulation of AsA metabolism by red and blue light (Bienger and Schopfer, 1970; Bartoli et al., 2009; Mastropasqua et al., 2012). There is a coaction between phytochrome and blue-light photoreceptors, as the former can absorb blue light and the latter can modulate responsiveness to the active form of phytochrome (Mohr, 1994; Mastropasqua et al., 2012).

### Continuous Light With a Low Red:Blue Ratio Improves AsA Accumulation by Promoting AsA Regeneration Rather Than Biosynthesis

To clarify the contribution of AsA biosynthesis and regeneration process to the promoting effect of blue light on AsA content, the gene expression levels and enzyme activities of several enzymes involved in these processes were investigated in the present study. In the AsA biosynthesis pathway, the transcript levels of *GMP*, *GME*, *GGP*, *GPP*, and *GLDH* were all upregulated by the 25R:75B treatment on day 3. Among these genes, *GGP* and *GLDH* presented the greatest and lowest responses to light treatments, respectively, which were 1.94 and 1.43-fold more expressed under 25R:75B compared to 75R:25B treatment. While the red:blue light ratio had no significant effect on GLDH activity (Figure 3). These results illustrated that the contribution of biosynthesis to the AsA increase under high ratio of blue light was limited, and they also demonstrated that, compared to *GMP*, *GME*, *GGP*, and *GPP*, GLDH expression and activity were not limiting, as reported by previous research (Alhagdow et al., 2007; Massot et al., 2013). Inconsistent changes of biosynthetic gene expression and GLDH activity had been proposed in several previous studies (Pignocchi et al., 2003; Mastropasqua et al., 2012). The mechanism driving this phenomenon must be complicated. Besides the posttranscriptional regulation, there are other mechanisms that could modulate GLDH activity directly, such as redox regulation (Bartoli et al., 2005; Leferink et al., 2009). Another result about biosynthesis process that needs to be noted was that *GGP* showed the greatest response to not only the red:blue light ratio but also to continuous light. A similar observation has been reported in Arabidopsis grown under continuous light (Yabuta et al., 2007). According to many previous studies, *GGP* was recognized as a key control gene in AsA metabolism, as it exhibited great differences among different environmental conditions, including different light conditions (Yabuta et al., 2007; Li et al., 2013).

For the AsA regeneration process, on the one hand, the increase in MDHAR, DHAR, and GR activities and gene transcription levels can theoretically promote AsA accumulation as they catalyze the reduction of DHA and MDHA. On the other hand, although the increase of APX activity consume more AsA, it also elevates AsA regeneration efficiency with the improvement of MDHAR and DHAR activities. In a previous study, *CitAPX3*, *CitchAPX*, *CitMDHAR1*, *CitMDHAR2*, *CitDHAR2*, *CitGR*, and *CitchGR* genes expression level along with AsA content were greater under blue LED light than dark conditions in citrus juice sacs (Zhang et al., 2015). Similar to the previous study, the expression levels of *APX1*, *APX2*, *MDHAR 1*, *MDHAR 2*, *MDHAR 3*, *DHAR 1*, *DHAR 2*, *GR 1*, and *GR 2* were all upregulated by the 25R:75B treatment on day 3. The significant higher activities of APX, MDHAR, DHAR, and GR under 25R:75B were observed subsequently. According to previous studies, increases in AsA content were often accompanied by increased APX, MDHAR, DHAR, and GR activities and gene expression levels, especially under some stress conditions (Mastropasqua et al., 2012; Gallie, 2013; Zha et al., 2019a). These results indicate that the maintenance of greater AsA levels under continuous light with a low red:blue ratio was attributed to the upregulated activities and gene expressions of enzymes involved in AsA regeneration rather than biosynthesis.

Notably, if we formulate conclusions only based on gene expression, we might mistakenly infer that AsA synthesis also contributes to the greater AsA levels induced by the continuous light with a higher ratio of blue light. In fact, during the process of the expression of a particular gene, the relationships among mRNA, protein content, and enzyme activity are quite complicated, and their correlations are often not obvious and not entirely recognized in plants (Veìlez-Bermuìdez and Schmidt, 2014; Liu et al., 2016; Szymanska et al., 2017).

### Regulation of Red:Blue Ratio on AsA Under Continuous Light Correlates With Oxidative Stress

In addition to AsA, the levels of several other antioxidants were also elevated by blue light, such as carotene (Ma et al., 2012), anthocyanins, and flavonoids (Li and Kubota, 2009; Ren et al., 2014). Furthermore, the enzymes involved in AsA regeneration are substantial components of enzymatic antioxidant system. This demonstrates that regulation of red:blue ratio on AsA correlates with oxidative stress under continuous light. Although continuous light (200 μmol⋅m^–2^⋅s^–1^, 15 days) didn’t induce leaf injury on lettuce in our previous study, it increased photo-oxidative pressure, which was characterized by enhanced ROS content and antioxidant activity (Zha et al., 2019b). Additionally, it had been reported that high ratio of blue light aggravated continuous light induced injury on tomato (Demers and Gosselin, 2002). In the present study, the H_2_O_2_ content presented a positive correlation with the ratio of blue light during the whole experiment. Thus AsA pool size, as well as the activities and gene expressions of APX, DHAR, MDHAR, and GR activity were activated by the high ratio of blue light to synergistically repress H_2_O_2_ generation and prevent cell oxidation. Photo-oxidative effect of continuous light with low red:blue ratio might due to the higher energy of blue light than red light at the same photon flux density. It has been reported that the gene expression and activity of AsA regeneration enzymes were activated by various environmental stresses (Haghjou et al., 2009; Gill and Tuteja, 2010), including photo-oxidative stress (Karpinski et al., 1999). Surprisingly, inconsistent with the H_2_O_2_ content, the MDA content was significantly higher under the 75R:25B treatment on day 12, indicating more severe membrane lipid peroxidation. This might be correlated with the greater amount and size of starch grains under 75R:25B treatment. Excessive starch accumulation under continuous light causes damage to the chloroplast structure (Aro and Valanne, 1979; Gestel et al., 2005). Thus, reactive oxygen species will leak from chloroplasts and cause severe membrane lipid peroxidation. In addition, it has been demonstrated that continuous light-induced leaf chlorosis was correlated with the starch accumulation (Haque et al., 2015).

### Regulation of Red:Blue Light Ratio on AsA Regeneration Related Gene Expression Depending on the Cell Compartment

Previous studies have confirmed that AsA recycling process occurs in several subcellular compartments, including mitochondria, chloroplasts, peroxisomes and cytosol (Koshiba, 1993; Yamaguchi et al., 1995; Jimenez et al., 1997), and each enzyme involved in AsA cycling has several isoenzymes (Smirnoff, 2011). In previous cases, different isoforms of APX, DHAR, MDHAR, and GR often showed different gene expression patterns or activities (Nishikawa et al., 2003; Gallie, 2013; Cocetta et al., 2014). In the present study, the transcription levels of genes encoding for different isoforms of these four enzymes were all up-regulated by 25R:75B on day 3. Different expression pattern of isoenzymes mainly emerged in the later experimental stage. According this, genes encoding different isoenzymes could be divided into two groups: chloroplast and others (cytosol, peroxisomal, and unidentified). Expression level of two chloroplast group genes (*MDHAR 1* and *GR 2*) under all treatments decreased sharply in the later experimental stage. By contrast, the cytosolic isoforms (*APX1* and *GR1*) presented greater responses to red:blue light ratio, meanwhile, the expression patterns of other unidentified isoforms were very similar with cytosolic isoforms. Nishikawa et al. (2003) also found transcription of AsA regeneration enzymes in harvested broccoli florets were inactivated in chloroplasts, but not in the cytosol, which might attribute to the excess generation of ROS in chloroplast under stress (Mano et al., 2001; Nishikawa et al., 2003). Moreover, H_2_O_2_ leaks from chloroplasts and peroxisomes into the cytosol, leading to the high demand of cytosol for AsA to protect the cells from oxidative damage (Nishikawa et al., 2003), oxidized AsA (MDHA and DHA) is then transported into the cytosol for reduction to AsA (Takahama, 2004). These demonstrated that AsA regeneration in cytosol takes on more and longer responsibility to defense stress than that in chloroplast. In addition, another interesting result in the present study is that expression levels of all genes except chloroplastic isoforms (*MDHAR 1* and *GR 2*) under the 50R:50B and 75R:25B treatments presented remarkable increases after day 3 and reached higher levels than those under the 25R:75B treatment. This demonstrated that the influence of the red:blue light ratio on AsA metabolism has the potential to reverse after 12 days of continuous light.

## Conclusion

In summary, a high ratio of red light was effective for enhancing the biomass and growth of hydroponic lettuce under continuous light, while the AsA pool size was elevated by a high ratio of blue light. According to the responses of gene expression and the activity of enzymes involved in AsA biosynthesis and regeneration, we concluded that the AsA regeneration process contributed more to AsA accumulation under continuous light with high ratio of blue light than biosynthesis. Since the high ratio of blue light has little influence on the activity of the final AsA synthase (GLDH), though it enhances the transcript level of genes involved in AsA biosynthesis. The response of the AsA metabolism to the blue light level at the gene expression level occurred earlier than the response at the enzymatic level. To have an integrative consideration, applying continuous light with a high ratio of red light for prophase cultivation and continuous light with a high ratio of blue light for preharvest irradiation will be conducive for both yield and quality.

## Data Availability Statement

The datasets generated for this study are available on request to the corresponding author.

## Author Contributions

WL was the recipient of funds. WL and LZ conceived the experiment. LZ, YZ, CZ, and MS prepared the plant materials, collected samples, and undertook experiments. LZ analyzed the data and prepared the manuscript. All authors contributed to the manuscript revision.

## Conflict of Interest

The authors declare that the research was conducted in the absence of any commercial or financial relationships that could be construed as a potential conflict of interest.

## Abbreviations

APX: ascorbate peroxidase
AsA: ascorbate
DHA: dehydroascorbate
DHAR: dehydroascorbate reductase
GGP: GDP-L-galactose phosphorylase
GLDH: L-galactono -1,4-lactone dehydrogenase
GME: GDP-D-mannose 3 ′,5 ′ -epimerase
GMP: GDP-D-mannose pyrophosphorylase
GPP: L-galactose -1-P phosphatase
GR: glutathione reductase
MDHAR: monodehydroascorbate reductase
T-AsA: total ascorbate.
