# Supplementary material for: Regulation of Ascorbate Accumulation and Metabolism in Lettuce by the Red:Blue Ratio of Continuous Light Using LEDs
Source: Front Plant Sci. 2020 May 29;11:704. doi: 10.3389/fpls.2020.00704 (PMC7272677; doi:10.3389/fpls.2020.00704)
Supplement: Supplementary file 1 [file Table_1.docx]

Supplemental Table S1 Nucleotide sequence of specific primers used for qRT-PCR in the experiment for the enzymes involved in the ascorbate metabolism.

| Gene | Forward primer (5’-3’) | Reverse primer (5’-3’) |
| --- | --- | --- |
| *GMP* | ATTGGTGACGGGTGTTTGAT | CACACATGCACATCCTCTCC |
| *GME* | TTGCAGGCACTACACCAAAG | ACCATCTCCCCACATTTCAA |
| *GGP* | GGAGCCTCCTTTTGCTTTCT | CGACCCTCGTTTAGTTGAGC |
| *GPP* | GAGCATAAAGGATCGGTGGA | GAACCCATGGACAAAGTTGG |
| *GLDH* | GAAGCAGAAGATCCGTCCTG | TACCTGAACAATGCCACCAA |
| *APX 1* (cytosolic) | TTCTATCAGTTGGCTGGTGTTG | TACTTGCCTCAAATGGTCGTT |
| *APX 2* | TGTATCAGCTTGCAGGTGTTGT | CCTTTGGTAGCATTTGGGAGT |
| *MDHAR 1* (chloroplastic/mitochondrial) | AGTTGGAGGTGGAAATGCTG | ACCTCCTGAACCCACACAAG |
| *MDHAR 2* | TGAAGTTGTATGGTGACATCAGAAG | AGATCGAAGGAACGCGAGTAG |
| *MDHAR 3* (peroxisomal) | AGAAAGGCGGAATCAAAGTCA | GCGGGAATAGAAGAATGGTAAG |
| *DHAR 1* | CTTGCCGAGAAGGGTGTTT | TTTGCCGTTGTGGATGAGA |
| *DHAR 2* | TGAGGTCTGTTGCAAAGCTG | CAACGGAACTTTCCCATTTG |
| *GR 1* (cytosolic) | TTCTGGGAGGCAAGAGAAGA | CCGCATGGTAACAAACTCCT |
| *GR 2* (chloroplastic) | AGGACGAGGAAAGATTGTGGA | GCAAACTCAAGGGCAATGTAA |
| *18S rRNA* | AAGCCCGATCCAGCAATAT | GGCGACTTTCACTTTCAACC |
